# Studies of *Anopheles gambiae *s.l (Diptera: Culicidae) exhibiting different vectorial capacities in lymphatic filariasis transmission in the Gomoa district, Ghana

**DOI:** 10.1186/1756-3305-3-85

**Published:** 2010-09-14

**Authors:** Hilaria Amuzu, Michael D Wilson, Daniel A Boakye

**Affiliations:** 1 Department of Parasitology, Noguchi Memorial Institute for Medical Research, University of Ghana, P.O. Box LG 581, Legon, Accra, Ghana; 2 WHO-TDR, 1211 Geneva 27, Switzerland

## Abstract

**Background:**

Two lymphatic filariasis endemic communities Mampong and Hwida in Ghana have been regularly monitored for impact on transmission after annual mass drug administration (MDA) with albendazole and ivermectin. After six MDAs even though the ABR for Mampong was 55883/person/year and that of Hwida was 2494/person/year, they both had ATPs of 15.21 infective larvae/person/year. Interestingly the human microfilaraemia levels had reduced significantly from 14% to 0% at Mampong and 12% to 3% at Hwida. In an attempt to understand this anomaly, we collected mosquitoes over a 5-month period using human landing catches to determine the species composition, the number of cibarial teeth, the lengths and widths of the cibarium and the cibarial dome of the vector populations.

**Results:**

Out of 2553 mosquitoes caught at Mampong, 42.6% were *An. gambiae *s.l. All 280 identified further by PCR were *An. gambiae *s.s (275 M and 5 S molecular forms). At Hwida, 112 mosquitoes were obtained; 67 (59.8%) were *An. gambiae *s.l, comprised of 40 (59.7%) *An. melas*, 24 (35.8%) *An. gambiae *s.s (17 and 5 M and S molecular forms respectively) and 3 (4.5%) unidentified. The mean number of teeth for *An. melas *was 14.1 (median = 14, range = 12-15), *An. gambiae *s.s., 15.7 (median = 15, range = 13-19) M form 15.5 (median = 15 range = 13-19) and S form 16 (median = 16, range 15-17). The observed differences in teeth numbers were significantly different between *An. melas *and *An. gambiae *s.s (p = 0.004), and the M form (p = 0.032) and the S form (p = 0.002).

**Conclusions:**

In this study, *An. gambiae *s.s was the main vector at Mampong and was found to possess significantly more cibarial teeth than *An. melas*, the principal vector at Hwida. We postulate that the different impact observed after 6 MDAs may be due to *An. gambiae *s.s exhibiting 'facilitation' at Mampong and at Hwida *An. melas *the main vector exhibits 'limitation'. Thus it may be necessary to compliment MDA with vector control to achieve interruption of transmission in areas where *An. melas *may exhibit limitation.

## Background

Lymphatic filariasis (LF) caused by infections with *Wuchereria bancrofti *is a debilitating disease which has adverse effects on productivity of infected persons and socioeconomic development of endemic countries [1]. The parasite is transmitted through the bite of infected mosquito species of various genera including, *Culex*, *Anopheles *and *Aedes*. In Ghana, members of the *An. gambiae *species complex and *An*. *funestus *are the principal vectors [2-5] although *An. pharoensis *has been implicated as a vector [3]. *Anopheles gambiae *s.l. in Ghana comprises *An. gambiae *s.s (which has two molecular forms; M and S), *An. melas *and *An. arabiensis *[6-8]. Unlike in Asia and East Africa, the *Culex *species in Ghana are refractory to the parasite [4].

An important determinant of transmission of *W. bancrofti *is the ability of the mosquito to ingest and support the development of microfilariae (mf) [9]. This ability is compromised when the mosquitoes possess cibarial armatures or 'teeth' in the foregut that lacerate ingested mf [10] thus reducing the number of mf that could develop. However the extent of reduction will depend on the number of teeth; the higher the number the more effective the armature should be at reducing mf numbers. According to Mcgreevy *et al*., [10] pharyngeal armatures, spines and papillae present in the foregut of mosquitoes may pose some threat to ingested mf but the most lethal structure in the foregut is the cibarial armature.

The laceration of mf during ingestion by the mosquito is independent of the mf density in the human host blood [11] but it is more pronounced at low mf densities. This results in the phenomenon termed 'facilitation' which is a positive feedback mechanism exhibited by some mosquito vectors. In facilitation, the number of ingested mf developing to infective stage (L3) increases as the number of mf ingested increases [12,13]. Such vectors are efficient mainly at high microfilaraemia levels. It is postulated that in areas where mosquito species exhibit facilitation, it should be possible to eliminate lymphatic filariasis by interrupting transmission with mass drug administration (MDA) alone. *Anopheles *mosquitoes possess cibarial armatures which lacerate ingested mf [11,10] and are reported to exhibit facilitation in the Gambia [14] and Papua New Guinea [15]. This observation forms the basis of the strategy of the Global Programme to Eliminate Lymphatic Filariasis (GPELF) that 5-6 annual rounds of MDA to all at-risk populations in areas where the vectors exhibit facilitation e.g. transmission of *W. bancrofti *by *Anopheles *species, will lead to elimination [14,16,17].

A study in northern Ghana gave the first indication that members of the *An. gambiae *s.l. could be exhibiting limitation [5] which is a negative feedback mechanism whereby the number of ingested mf developing to infective stage (L3) decreases as the number of mf ingested increases [12,13]. However the authors of this study pooled the vectors *An. gambiae *s.l. and *An. funestus *together in the analysis to arrive at this conclusion because of the few numbers of mosquitoes obtained. Since then the individual species have not been studied further to determine vector competencies at low microfilaraemia levels.

Annual MDA has been ongoing in the Gomoa District, Ghana since 2001 and entomological monitoring of the impact has been conducted at eight sentinel communities in the District. After 6 rounds of MDA, the overall microfilariae (mf) prevalence in the human population had decreased from 14% in 2001 to 0% in 2007 in the district (Boakye DA, unpublished report to WHO/TDR). However, this general reduction was not observed at some of the sites notably at Hwida but markedly so at another site, Mampong. The entomological monitoring at the two sites showed that at baseline, Mampong had a human microfilaraemia level of 14% with *An. gambiae *s.l annual biting rate (ABR) of 56,164 bites/person/year and an ATP (annual transmission potential) of 129.29 infective larvae/person/year. At Hwida comparative values were *An. gambiae *s.l ABR of 411/person/year with only infected mosquitoes (0.074) and none infective, and therefore an ATP of zero and human microfilaraemia of 12%. After six MDAs the ABR, ATP and human microfilaraemia levels were 55,883/person/year, 15.21 infective larvae/person/year and 0% respectively at Mampong. At Hwida the corresponding values were 2494/person/year, 15.21 infective larvae/person/year and 3% respectively. That the relatively small population size of *An*. *gambiae *s.l at Hwida was responsible for maintaining transmission there while the opposite held true at Mampong suggested to us that the two *An. gambiae *s.l populations could be different in their vectorial competencies with that at Hwida being more efficient at low level microfilaraemia i.e. exhibiting limitation. Earlier species identification during the monitoring period had indicated a mixture of *An. gambiae *s.s. and *An. melas *at Hwida and only *An. gambiae *s.s. at Mampong but these were not studied further.

This study therefore investigated the sibling species composition of *An. gambiae *complex and their cibarial armatures in terms of teeth numbers, and the sizes of the cibarium and the dome as surrogate determinants of their vectorial competencies.

## Methods

### Study sites

The endemic communities; Mampong (05° 24' N, 00° 36' W) and Hwida (05° 15' N, 00° 48' W) are located in the Gomoa District, which is approximately 80 km west of Accra, the capital city of Ghana.

Mampong is located near the Okyereko Irrigation Scheme which was created for rice farming. The irrigation scheme provides suitable breeding grounds for *Anopheles *mosquitoes [7] leading to a high density of *Anopheles *in the area. The estimated population size of Mampong is 960 inhabitants. Hwida is located on the coast of the Atlantic Ocean with an estimated population size of 456 inhabitants. A large man-made pond at Hwida, which serves as a source of water for the inhabitants, is a major feature of its landscape. The two communities are approximately 28 km apart and they both have the same vegetation which is coastal savannah. Previous studies have implicated *An*. *gambiae *s.l. and *An. funestus *as the vectors of LF in the Gomoa District [2,3].

### Mosquito sampling

Mosquito surveys by hourly indoor human landing catches (HLC) were carried out simultaneously in the two communities from 1800 H to 0600 H a day each month from August to December 2007 by trained volunteers after obtaining ethical approval from the Institutional Review Board of the Noguchi Memorial Institute for Medical Research and consent from the volunteers. The sampling period was selected to correspond to the highest mosquito breeding season in the area, based on the previous sampling in the area. A household was selected randomly and a room used for collection each month; thus a total of 5 rooms were sampled per community. The mosquitoes were collected by two volunteers per community. Each mosquito was initially identified morphologically to *An. gambiae *s.l. [18,19]. The head of each *An. gambiae *s.l. mosquito was then separated from the rest of the body and stored in 1.5 ml Eppendorf tubes containing silica gel until needed for the cibarial armature studies. The thorax and abdomen together were similarly kept in separate Eppendorf tubes until needed for the molecular identifications to sibling species and the M and S molecular forms.

### PCR identification of *An. gambiae *species and *An. gambiae *s.s. molecular forms

DNA extraction from homogenised thorax and abdomen tissue used the method of Collins *et al*. [20] and the PCR identification to species used the method of Scott *et al*. [21]. A restriction digest of the PCR product of identified *An. gambiae *s.s was then carried out to determine the molecular forms [22].

### Cibarial armature

The head of each mosquito was placed in an appropriately labelled 0.5 ml Eppendorf tube and three drops of clearing medium (equal volumes of chloral hydrate and phenol) was added and kept in the dark for 7 days. The head was removed and placed on a microscope slide and a drop of Puri's medium added [23]. In most instances, the position of the mount was manipulated until the ideal position that facilitated counting of the cibarial teeth was obtained before covering with a cover slip. The cibarial armature was then viewed under a compound microscope at 400× magnification. The number of cibarial teeth of each head was then counted and recorded. A 10 μm (micrometer) eye piece gauge was used to measure the length and width of the cibarium and the cibarial dome for each mounted sample.

### Statistical analysis

Univariate functions in SPSS version 11.5 for Windows™ (SPSS Inc, Cary, USA) were used to calculate the means, median, range and the standard deviations. Analysis of variance (ANOVA) was then used to test the significance of the comparative values obtained (significance: p < 0.05).

## Results

A total of 2553 mosquitoes were collected at Mampong of which 1087 (42.6%) were *An. gambiae *s.l. Other mosquito species obtained were *Culex spp*. (29.9%), *Mansonia spp*. (23.9%), *An. pharoensis *(2.7%), *An. funestus *(1.6%) and *Aedes spp*. (0.1). Of the 1087 *An. gambiae *s.l collected, 290 were further examined with PCR and 280 (96.6%) were all identified as *An. gambiae *s.s made up of 275 (98.2%) M and 5 (1.8%) S molecular forms. Ten (3.4%) specimens failed to amplify.

A total of 112 mosquitoes were collected at Hwida and 67 (59.8%) were *An. gambiae *s.l. The other mosquito species obtained were distributed as follows; *Culex spp*. (29.5%), *Mansonia spp*. (4.5%), *An. pharoensis *(0.9%), *An. funestus *(4.5%) and *Aedes spp*. (0.9%). The 67 *An. gambiae *s.l. comprised of 40 (59.7%) *An. melas*, 24 (35.8%) *An. gambiae *s.s and 3 (4.5%) failed to amplify. Of the 24 *An. gambiae *s.s. 17 (71%) were M molecular form and 7 (29%) were S molecular form.

**Figure 1:**
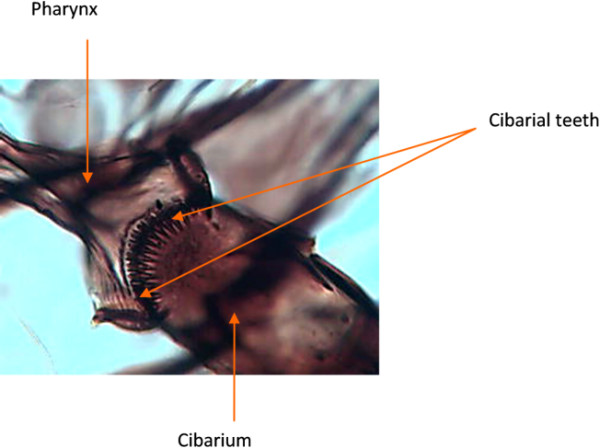
**Cibarial armature of *Anopheles gambiae *s.l**.

A total of 71 *An. gambiae *s.l. (35 *An. melas *and 36 *An. gambiae *s.s) were processed for the cibarial armature studies. However, the cibarial armatures of only 17 *An. gambiae *s.s. (11 M and 6 S molecular forms) and 13 *An. melas *could be read. A typical example of the cibarial armature of *An. gambiae *s.l is shown in figure 1. The number of teeth counted for all the species were within 12 to 19 (Table 1). The observed difference in teeth numbers was significantly fewer (p = 0.004) in *An. melas *than in *An. gambiae *s.s. There was, however, no significant difference in the number of teeth of the M and S molecular forms of *An. gambiae *s.s (p = 0.503). The number of teeth of *An. melas *was, however, significantly fewer than *An. gambiae *M molecular form (p = 0.032) and S molecular form (p = 0.002).

**Table 1:** Number of cibarial teeth, length and width of cibarial dome and cibarium of *An. gambiae *s.l

Species	Median teeth number (range)	Mean number of teeth	SD (Range)	Mean length of dome (μm)	SD (Range)	Mean width of dome (μm)	SD (Range)	Mean length of cibarium (μm)	SD (Range)	Mean width of cibarium (μm)	SD (Range)
*An. gambiae *s.s	15 (13-19)	15.7	± 1.54(13-19)	1.04	± 0.12(0.8-1.2)	1.73	± 0.21(1.2-2.4)	4.12	± 0.48(3.2-4.8)	2.32	± 0.31(1.6-2.8)
M form	15 (13-19)	15.5	± 1.57(13-19)	1.05	± 0.13(0.8-1.2)	1.73	± 0.24(1.2-2.4)	4.16	± 0.26(4.0-4.6)	2.40	± 0.28 (1.8-2.8)
S form	16 (15-17)	16.0	± 1.51(15-17)	1.04	± 0.08(1.0-1.2)	1.73	± 0.10(1.6-1.8)	4.10	± 0.53 (3.2-4.8)	2.07	± 0.30 (1.8-2.4)
*An. melas*	14 (12-15)	14.1	± 1.56(12-15)	1.05	± 0.18(0.8-1.4)	1.70	± 0.13(1.4-1.8)	3.91	± 0.76 (2.8-5.0)	2.24	± 0.26 (1.8-2.8)

There was no significant difference in the lengths and widths of both the cibarial dome and cibarium of *An. melas*, M and S molecular forms of *An. gambiae *s.s (p > 0.05).

## Discussion

The ability of mosquito genera to transmit filarial parasites, particularly at low microfilaria density vary and three density-dependent processes; limitation, facilitation and proportionality have been described to explain them [14]. "Limitation" describes the situation where vectors are efficient even at very low parasite densities and "facilitation" for the vectors that are efficient mainly at high microfilaraemia levels. *Anopheles *species are considered to exhibit the process of facilitation [14,16,17] and therefore it is assumed that low level microfilaraemia resulting from MDA would lead to interruption of transmission and elimination of lymphatic filariasis in anopheline transmission areas.

One factor that has been reported to influence the efficacy of vector species is the cibarial armature which is known to have lethal effect on ingested filarial parasites [10]. The fewer the ingested parasites the more pronounced the laceration effect by the cibarial armature since most will be effectively damaged. However as the ingested parasite numbers increase, this effect decreases since some parasites will mask the teeth thereby allowing others to pass through unscathed [24]. McGreevy *et al*. [10] surmised that the extensive variations observed in the size, shape and number of cibarial teeth of mosquitoes could be determinants of their vectorial efficiency. With this reasoning cibarial armature with low teeth numbers functions less as a barrier therefore vectors possessing this feature should transmit effectively even at low densities of ingested parasites.

The number of teeth reported by this study for the *An. gambiae *species falls within the range of 12-20 that was reported for *An. gambiae *s.l [18]. However, this study did not distinguish the sibling species. In a later study, Bryan and Southgate [11] observed that *An. melas *destroyed relatively fewer numbers of microfilariae than *An. gambiae *s.s but did not give any reasons for this. In the present study we show that *An. melas *possess significantly lower number of teeth than both M and S forms of *An. gambiae *s.s. That *An. melas *possessed significantly lower number of cibarial teeth than *An. gambiae *s.s (the molecular forms included), is important information and explains for the first time the possibility that differences may exist in the vectorial competencies within the *An. gambiae *sibling species complex in West Africa in the transmission of *W. bancrofti*. The fewer number of cibarial teeth in *An. melas *as observed may therefore make this species a more efficient vector particularly at low level microfilaraemia and thus exhibit limitation, while *An. gambiae *s.s. on the contrary exhibit facilitation. This difference in vector capabilities at low microfilaraemia between the two vector species could therefore be relevant in explaining the different impact of the six rounds of MDA on transmission observed in the two communities being significantly reduced at Mampong but relatively unchanged at Hwida.

The results obtained by this study also add to the body of evidence that, at least in Ghana, not all *Anopheles *species may exhibit the process of facilitation. Moreover, that these two phenomena i.e. limitation and facilitation occur in communities that are in terms of distance very close (28 km) suggests that vectorial systems at the local level should be taken into account if LF elimination is considered. Vector control should therefore be considered in addition for areas where the principal vectors exhibit limitation as is found in the current study for *An. melas*.

Although *An. funestus *is a known vector of LF in Ghana, it was found in very low numbers and was therefore not considered to play any significant role to influence transmission in the two studied communities. However, in light of the results obtained it may be worthwhile to study this species especially in northern Ghana for example where it is a major vector of LF and limitation is suspected [5] but *An. melas *has not been reported there.

## Conclusion

We observed from this study that *An. gambiae *s.s possess significantly more cibarial teeth than *An. melas*. This could explain the different impact observed after 6 MDAs at Mampong and at Hwida and the reported differences in the vectorial competencies within the *An. gambiae *sibling species complex in West Africa in the transmission of *W. bancrofti*. We suggest that an understanding of the local vectorial system may therefore be necessary in areas where MDA alone is not achieving the goals of LF elimination.

## Competing interests

The authors declare that they have no competing interests.

## Authors' contributions

All the authors have contributed significantly to this study. DAB and MDW contributed intellectually to the conceptualization, design of both the study and manuscript preparation. HA carried out the laboratory and field studies and manuscript preparation. All authors read and approved the final manuscript.

## References

[B1] World Health Organization Annual report on lymphatic filariasis 2001. Geneva 2002

[B2] Dunyo SK Appawu M Nkrumah FK Baffoe-Wilmot A Pedersen EM Simonsen PE Lymphatic filariasis on the coast of Ghana Trans R Soc Trop Med Hyg 1996 90 634 638 10.1016/S0035-9203(96)90414-9 9015499

[B3] Dzodzomenyo MD Dunyo SK Ahorlu CK Coker WZ Appawu MA Pedersen EM Simonsen PM Bancroftian filariasis in an irrigation project community in southern Ghana Trop Med Int Health 1999 4 13 18 10.1046/j.1365-3156.1999.00354.x 10203168

[B4] Appawu MA Dadzie SK Baffoe-Wilmot A Wilson MD Lymphatic filariasis in Ghana: entomological investigation of transmission dynamics and intensity in communities served by irrigation systems in the Upper East Region of Ghana Trop Med Int Health 2001 6 511 516 10.1046/j.1365-3156.2001.00737.x 11469943

[B5] Boakye DA Wilson MD Appawu MA Gyapong J Vector competence for *Wuchereria bancrofti *of the *Anopheles *populations in the Bongo district of Ghana Ann Trop Med Parasitol 2004 98 5 501 508 10.1179/000349804225003514 15257800

[B6] Appawu MA Baffoe-Wilmot A Afari EA Nkrumah FK Petrarca V Species composition and inversion polymorphism of the *Anopheles gambiae *complex in some sites of Ghana, West Africa Acta Trop 1994 56 15 23 10.1016/0001-706X(94)90036-1 8203292

[B7] Okoye PN Wilson MD Boakye DA Brown CA Impact of the Okyereko irrigation project in Ghana on the risk of human malaria infection by *Anopheles *species (Diptera: Culicidae) Afr Entomol 2005 13 2 249 253

[B8] Yawson AE Weetman D Wilson MD Donnelly MJ Ecological zones rather than molecular forms predict genetic differentiation in the malaria vector *Anopheles gambiae *s.s. in Ghana Genetics 2007 175 751 761 10.1534/genetics.106.065888 17110481 PMC1800615

[B9] Bryan JH Southgate VA Factors affecting transmission of *Wuchereria bancrofti *by *Anopheles *mosquitoes. 1. Uptake of microfilariae Trans R Soc Trop Med Hyg 1988 82 128 137 10.1016/0035-9203(88)90286-6 3051542

[B10] McGreevy PB Bryan JH Oothuman P Kolstrup N The lethal effects of the cibarial and pharyngeal armatures of mosquitoes on microfilariae Trans R Soc Trop Med Hyg 1978 72 361 368 10.1016/0035-9203(78)90128-1 30190

[B11] Bryan JH Southgate VA Factors affecting transmission of *Wuchereria bancrofti *by *Anopheles *mosquitoes. 2. Damage to ingested microfilariae by mosquito foregut armatures and development of filarial larvae in mosquitoes Trans R Soc Trop Med Hyg 1988 82 138 145 10.1016/0035-9203(88)90288-X 3051543

[B12] Bain O Transmission des filarioses. Limitation des passages de microfilaires ingérées vers l'hémocèle des vecteurs; interprétation Ann Parasitol Hum Comp 1971 46 613 631 5170431

[B13] Brengues J Bain O Passage des microfilaires de l'estomac vers l'hemocele du vecteur, dans les couples *Wuchereria bancrofti - Anopheles gambiae *A, *Wuchereria bancrofti - Aedes aegypti *et *Setaria labiatopapillosa - Aedes aegypti* Cah O R S T O M Ser Entomol Med Parasitol 1972 10 235 249

[B14] Southgate BA Bryan JH Factors affecting transmission of *Wuchereria bancrofti *by anopheline mosquitoes. 4. Facilitation, limitation, proportionality and their epidemiological significance Trans R Soc Trop Med Hyg 1992 86 523 530 10.1016/0035-9203(92)90096-U 1475823

[B15] Bockarie MJ Tisch DJ Kastens W Alexander ND Dimber Z Bockarie F Ibam E Alpers MP Kazura JW Mass treatment to eliminate filariasis in Papua New Guinea N Engl J Med 2002 347 1841 1848 10.1056/NEJMoa021309 12466508

[B16] Weber RH Can anopheline-transmitted filariasis be eradicated? J Trop Med Hyg 1991 94 241 244 1880825

[B17] Snow LC Bockarie MJ Michael E Transmission dynamics of lymphatic filariasis: vector-specific density dependence in the development of *Wuchereria bancrofti *infective larvae in mosquitoes Med Vet Entomol 2006 20 261 272 10.1111/j.1365-2915.2006.00629.x 17044876

[B18] Gillies MT De Meillon B The Anophelinae of Africa South of the Sahara (Ethiopian Zoogeographical Region) 1968 South African Institute for Medical Research

[B19] Gillies MT Coetzee M A supplement to the Anophelinae of Africa South of the Sahara (Afrotropical Region) 1987 South African Institute for Medical Research

[B20] Collins FH Mendez MA Rasmussen MO Mehaffey PC Besansky NJ Finnerty V A ribosomal RNA gene probe differentiates member species of the *Anopheles gambiae *complex Am J Trop Med Hyg 1987 37 37 41 2886070 10.4269/ajtmh.1987.37.37

[B21] Scott JA Brogdon WG Collins FH Identification of single specimens of the *Anopheles gambiae *complex by the polymerase chain reaction Am J Trop Med Hyg 1993 49 4 520 529 8214283 10.4269/ajtmh.1993.49.520

[B22] Fanello C Santolamazza F della Torre A Simultaneous identification of species and molecular forms of the *Anopheles gambiae *complex by PCR-RFLP Med Vet Entomol 2002 16 461 464 10.1046/j.1365-2915.2002.00393.x 12510902

[B23] Smart J Jordan K Whittick RJ Insect of Medical Importance 1965 4 Adlen Press: Oxford

[B24] Duerr H Dietz K Schulz-Key H Büttner DW Eichner M Determinants of the eradicability of filarial infections: a conceptual approach Trends Parasitol 2005 21 88 96 10.1016/j.pt.2004.11.011 15664532

